# Insights Into Lung Cancer Immune-Based Biology, Prevention, and Treatment

**DOI:** 10.3389/fimmu.2020.00159

**Published:** 2020-02-11

**Authors:** Sara Saab, Hussein Zalzale, Zahraa Rahal, Yara Khalifeh, Ansam Sinjab, Humam Kadara

**Affiliations:** ^1^Department of Biochemistry and Molecular Genetics, Faculty of Medicine, American University of Beirut, Beirut, Lebanon; ^2^School of Medicine, American University of Beirut, Beirut, Lebanon; ^3^Department of Translational Molecular Pathology, University of Texas MD Anderson Cancer Center, Houston, TX, United States

**Keywords:** lung cancer, non-small cell lung cancer, anti-tumor immunity, immunogenomics, immunotherapy, lung premalignancy

## Abstract

Lung cancer is the number one cause of cancer-related deaths. The malignancy is characterized by dismal prognosis and poor clinical outcome mostly due to advanced-stage at diagnosis, thereby inflicting a heavy burden on public health worldwide. Recent breakthroughs in immunotherapy have greatly benefited a subset of lung cancer patients, and more importantly, they are undauntedly bringing forth a paradigm shift in the drugs approved for cancer treatment, by introducing “tumor-type agnostic therapies”. Yet, and to fulfill immunotherapy's potential of personalized cancer treatment, demarcating the immune and genomic landscape of cancers at their earliest possible stages will be crucial to identify ideal targets for early treatment and to predict how a particular patient will fare with immunotherapy. Recent genomic surveys of premalignant lung cancer have shed light on early alterations in the evolution of lung cancer. More recently, the advent of immunogenomic technologies has provided prodigious opportunities to study the multidimensional landscape of lung tumors as well as their microenvironment at the molecular, genomic, and cellular resolution. In this review, we will summarize the current state of immune-based therapies for cancer, with a focus on lung malignancy, and highlight learning outcomes from clinical and preclinical studies investigating the naïve immune biology of lung cancer. The review also collates immunogenomic-based evidence from seminal reports which collectively warrant future investigations of premalignancy, the tumor-adjacent normal-appearing lung tissue, pulmonary inflammatory conditions such as chronic obstructive pulmonary disease, as well as systemic microbiome imbalance. Such future directions enable novel insights into the evolution of lung cancers and, thus, can provide a low-hanging fruit of targets for early immune-based treatment of this fatal malignancy.

## Introduction

Cancer is a collection of diseases driven by genetic and epigenetic aberrations. In the classical sense, cancer pathogenesis is explained by mutations affecting proto-oncogenes and tumor suppressors, a paradigm that has proven rather simplistic particularly with the emergence of host immune deregulation as an important hallmark in cancer pathogenesis (1). The second most common cancer with the highest cancer mortality rate across both sexes is lung cancer, whose poor prognosis can be partially attributed to the scarcity of early detection strategies (2). With a strong need to develop screening tools for early markers signifying the development of lung cancer, as well as devise new treatment strategies to target the disease at its earliest stages, it is not surprising that lung cancer represents one of the most heavily studied cancers in immune–oncology.

Lung cancers are classified into two main histological types, small cell lung cancer (SCLC) and non-small cell lung cancer (NSCLC), with the latter further subdivided into multiple histologically and molecularly variant subtypes (3, 4). SCLCs are aggressive lung tumors that are often caused by smoking and encompass 15–20% of all primary lung cancers (3, 5). *MYC* gene amplifications and paraneoplastic syndromes are common in SCLC (5, 6). NSCLC can be divided into four subtypes: lung adenocarcinoma (LUAD), lung squamous cell carcinoma (LUSC), large cell carcinoma, and bronchial carcinoid tumor. Among these, LUAD is the most prevalent subtype of NSCLC, and the most common primary lung tumor overall. The malignancy, which frequently arises among female non-smokers, adopts a histologically glandular pattern with activating mutations affecting driver genes such as *KRAS, EGFR*, and *BRAF*, as well as *ALK* fusions and other genetic alterations (4).

Ideally, the immune system has the potential to monitor, recognize, and destroy malignant cells. However, tumors evolve several mechanisms to evade host immune-mediated surveillance and destruction. These include expansion of a local immunosuppressive microenvironment, induction of dysfunctional T cell signaling, and upregulation of inhibitory immune checkpoints which serve, under non-malignant conditions, to keep the immune system in check by preventing an indiscriminate attack against self-cells (1). This knowledge prompted the idea of tweaking the immune system of tumors, and later premalignant lesions, using immune-based therapies, to intercept malignant progression at multiple stages. Contemporary modalities of immunotherapy focus on harnessing these mechanisms to restore a competent anti-tumor host immunity. While early attempts were based on treating patients with interleukin (IL)-2 or interferon (IFN)-α to elicit a Th1 cell mediated immune response, T cells were the focus of later attempts which range from culture and reinfusion of tumor infiltrating lymphocytes (TIL), to T cell receptor (TCR) engineering, and the production of chimeric antigen receptors (CAR) that possess elements of both B and T cell receptors (7, 8). Later pioneering work introduced immune checkpoint blockade (ICB), a tumor intervention that re-activates the intrinsic antitumor immune response by blocking inhibitory immune receptors expressed on the surface of cancer cells or immune cells within the cancer microenvironment (9, 10). ICB remains, thus far, the most promising immunotherapeutic avenue for a number of cancers, as it actively targets the compromised milieu rather than the tumor itself. However, not all cancers have shown durable responses to immunotherapeutic intervention, whereby a number of cancers were described as being more efficiently “hidden” from host immune surveillance than others, or so-called immune “silent,” or “cold” (11, 12). These observations revealed a gap in our knowledge of the immune-biology of cancers, and sparked the emergence of a field in immuno-oncology that centers on delineating the immune changes during the pathogenesis of premalignant lesions and advanced tumors, in order to derive potential targets for screening, treatment, and even prediction of response to immunotherapies such as ICB.

This review summarizes current advances in immunotherapy and the current state of knowledge of lung cancer immune biology, with a particular focus on early-stage disease including premalignancy. It also uncovers the immunogenomic mechanisms behind the variable response of lung tumors to immunotherapy, with a focus on understanding naïve tumor immune biology and its role in modulating host microbiome particularly at the earliest stages of tumor pathogenesis. We then highlight the potential translational role of immunotherapy in early management of the disease.

## Malignant Immune Biology

Understanding the interaction of the tumor with its microenvironment, collectively referred to as the tumor microenvironment (TME), is a complex endeavor that challenges our understanding of the basic paradigms of innate control and adaptive immunity in the non-malignant setting. To understand how the tumor hijacks the host immunity and reprograms the TME leading to loss of immunological control, we will briefly review known mechanisms of immunosurveillance.

The role of the immune system is to eradicate foreign antigens, namely microbes and mutated self-cells, all while maintaining a state of homeostasis by sparing normal/self-tissue. This is mediated by an interaction between components of the adaptive and the innate immune system (13, 14). Activation of cells in the adaptive immune system composed of T cells and B cells produces a delayed yet specific immune response (13, 14). This is a highly orchestrated process dictated by multiple cues from the innate immune system, the frontline response activated following antigen exposure and, contrary to adaptive immunity, it is not programmed to produce specific cytolytic molecules. Conceptually, the current model posits that activation of an immune-mediated adaptive host defense relies on a “three-signal activation” between a T cell and an antigen presenting cell (APC) (1). The first interaction, “signal one”, occurs between a CD8 or CD4 molecule on the surface of the cytotoxic and helper T cells, respectively, and the non-peptide binding regions on the major histocompatibility complex proteins (MHC) class I or II molecule, respectively (1). The TCR also recognizes the antigen presented on the APC's MHC molecule. The second signal involves the binding of CD28, the prototypical co-stimulatory molecule found on T cells, to either CD80 or CD86 on APCs, which in turn activates the third signal: effector cytokine production by the APC (1). The three signals enable full activation of T cells and promote their clonal expansion. The triad is also the underlying mechanism for our growing understanding of tumor immune biology, immune evasion, and the clinical milestones in cancer immunotherapy.

### Tumor Immunosurveillance and Tumor Antigens

Tumor immunosurveillance is defined by the ability of immune cells to recognize and destroy occult cancerous cells (11, 15–17). This process is accomplished by both the innate and adaptive immune systems, though the latter plays a more prominent role (18), and this is aided by cancer-unique antigens that can be recognized by the immune system. One group of antigens are known as tumor associated antigens (TAA) which are antigens that are overexpressed in cancer cells but can also be found on normal tissues (19, 20). These include CD19, PRAME, MAGE, ERBB2, p53, and L2A5 (20–30). Studies have shown that tumors possessing these antigens are more likely to induce tolerance and are less responsive to ICB (further explained below) (22, 23). In addition, they induce more autoreactivity when targeted by adoptive cell therapy (ACT) (31–36).

**Figure 1:**
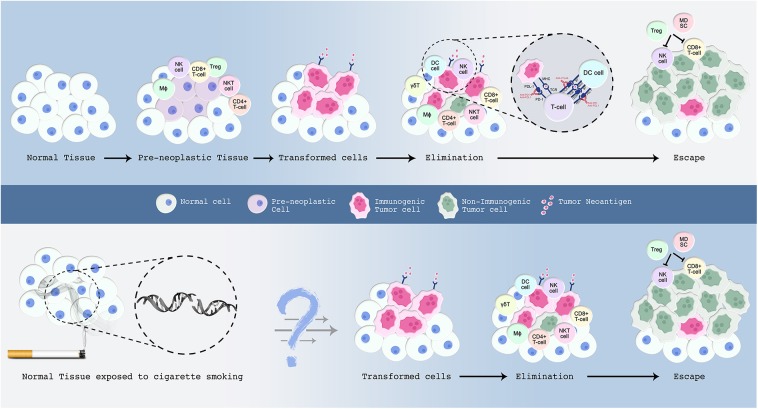
A proposed model for the malignant transformation of normal tissue with emphasis on the immune microenvironment. The events underlying this process are explained in the text. Normal cells are in blue; preneoplastic cells in violet; transformed cells in pink; and malignant cells in green. Upper panel: Normal cells accumulate somatic mutations in driver genes leading to the formation of premalignant cells. Those preneoplastic cells attract the immune system, wherein cells from both the innate and adaptive immune system infiltrate the tissue. Certain tumor cells evolve several mechanisms to evade host immune-mediated surveillance and destruction. Clinical inhibition of immune-checkpoints blocks checkpoint inhibitory action and re-activates the immune system to launch an attack on tumor cells. Lower panel: Smoking induces an extensive mutational repertoire leading to the formation of transformed cells. Many of the immune molecules and cells that participate in the elimination phase have been characterized, but future work is required to determine their exact sequence of action. In addition, further studies are warranted to understand the sequence of events that render a subset of smokers more prone to develop lung cancer compared to others who do not develop this malignancy throughout their lifetime.

Another group of antigens are collectively termed tumor specific antigens (TSA) which are as name implies unique to tumors (19, 37). TSAs result from the accumulation of mutations within a tumor cell line (19, 37). A special category of these antigens comprises neoantigens, which are present on MHC molecules (38, 39) (Figure 1). Surprisingly, neoantigens are unique to the patient rather than to the tumor, and are often downregulated in tumors, suggesting that tumors evade immune destruction (40–45). These properties make neoantigens suitable targets for personalizing cancer vaccines and ACT (46, 47). Earlier work studying the immune landscape of different cancers based on specific signatures that tell of immune function explains that neoantigen load is correlated to CD8+ T cells, M1 macrophages, CD4+ T cells, and lower T regs (48, 49). Later, and as explained in further detail below, neoantigens were shown to be important determinants of a response to ICB.

### Immune Checkpoints

Investigating the TME has also led to the discovery of a set of molecules termed “immune checkpoints” that can alter the immune system's ability to recognize malignant cells. Immune checkpoints comprise a set of receptors on the surface of activated T cells (34, 50). By interfering with the activation of T cells, immune checkpoint molecules ensure immune homeostasis and self-tolerance under “normal” physiologic conditions whereby collateral tissue damage is to be avoided, but contribute to tolerance of tumor cells by pushing the “brakes” on host immune activation (51, 52). In malignancy, clinical inhibition of these checkpoints releases those inhibitory “brakes,” blocks checkpoint inhibitory action and re-activates the immune system to launch an attack on tumor cells. This mechanism has been described with more than 20 known immune checkpoint modulators, among which are LAG-3, TIM3, TIGIT, as well as programmed cell-death receptor 1 (PD-1) and cytotoxic T lymphocyte associated protein- 4 (CTLA-4) which are described in further detail below (53, 54).

One way in which tumor cells mediate a checkpoint, or “brake” on T cell activation and thus anti-tumor immunity, is by expressing CTLA-4, a B7 ligand and an inhibitory homolog of CD28 (55) (Figure 1). Much like CD28, CTLA-4 is upregulated following antigen presentation (34, 55). It competes with CD28 for binding to CD80 (B7-1 ligand) or CD86 (B7-2 ligand), and thereby dampens the T cell-mediated response allowing malignant cells to evade immune destruction (51, 55). In the non-pathogenic setting, CTLA-4's constitutive expression on regulatory T cells (T regs) serves a key role in immune tolerance, a regulatory mechanism that prevents the formation of self-reactive T cells that are capable of inducing autoimmune diseases in the host (56). Indeed, CTLA-4 knockout mice develop lymphoproliferative disorders including multiorgan infiltration, tissue damage, and autoimmune diseases which ultimately leads to their death less than a month after birth (56–58). The magnitude and the breadth of stimulatory molecules in comparison to inhibitory counterparts, as exemplified here by a balance of CD28 and CTLA-4-derived signals, is critical to T cell activation and tolerance, and represents a window of opportunity for clonal selection during tumor evolution (54).

PD-1 engagement with its ligands (PD-L1 or PD-L2) constitutes a major immune checkpoint axis that regulates self-tolerance and contributes to the maintenance of immune homeostasis (59, 60). PD-1 is usually expressed on the surface of activated T cells, macrophages, B cells, and NK cells whereby its expression is predominantly upregulated in response to chronic antigen encounter as seen in cancer or chronic viral infections (61) (Figure 1). Along with the upregulation of other checkpoint molecules, such as TGIT, LAG-3, and Tim-3, PD-1 upregulation signifies immune adaptation to chronic stimulation and therefore leads to the attenuation of the immune response (61). Furthermore, it has been shown that PD-1 expression is elevated upon the secretion of type I and type II IFN from the tumor stroma (62, 63). This is supported by the fact that prolonged IFN-γ signaling in mouse models drives resistance to PD-1 blockade (64, 65). PD-1's engagement with its ligand PD-L1, found on tumor cells, TIL, APC, endothelial, and epithelial cells, further dampens the apoptotic pathway, and induces anergy as well as T cell depletion (51, 55, 66–69). The expression of PD-L2, another ligand for PD-1, is limited to dendritic cells (DCs) and macrophages (51, 67). Accumulating knowledge suggests that lung tumors overexpress the immunosuppressive protein, PD-L1, and inhibiting this pathway has led to durable benefit in a subset of advanced-stage NSCLC patients (69, 70).

## Immunotherapies Targeting Lung Cancer

Despite promising advances in conventional and targeted therapies seen across multiple cancers, lung cancer prognosis remains dismal, with a low 5-year survival rate of 18% in the US, mostly due to an advanced stage at diagnosis across the majority of lung cancer patients (2). Clinical trial results seen in response to immunotherapies, however, were an enormous leap in the field of lung cancer treatment. This led to US Food and Drug Administration's (FDA) approval of several immunotherapeutic options for lung cancer patients, including some as first-line therapy. The mechanisms behind the mode of immunomodulation mediated by multiple immune-based therapies is summarized below, with a focus on successful and promising options for lung cancer subtypes.

### Adoptive Cell Therapy

Adoptive cell therapy is a form of immunotherapy that involves expansion of patient-derived lymphocytes *ex vivo* before reinfusing them back in the patient, based on the rationale that proliferation of tumor-reactive lymphocytes is made possible when carried out away from the suppressive effects of the TME (71). The earliest documented study in the field was conducted on patients with various end stage tumors in which standard therapy had failed. Researchers cultured lymphocytes obtained from patients' peripheral blood with IL-2 to generate so-called lymphokine activated cells or LAK cells (72). LAK cells were subsequently reinfused in the patients along with multiple doses of IL-2 (72). Eleven out of 25 patients diagnosed with melanoma, LUAD, colorectal cancer, or renal cancer showed partial remission with one melanoma patient showing a complete response (72). The results potentiated ACT-based immunotherapy for the treatment of metastatic tumors and paved the way for the investigation of other forms of immunotherapy.

Tumor infiltrating lymphocytes (TILs) are a special group of immune cells that can be used in ACT. They are a group of lymphocytes that infiltrate the tumor microenvironment, owing to their heightened ability to detect cancer antigens and respond by releasing pro-inflammatory cytokines (73). TILs collected from surgically resected tumors were cultured *ex vivo*, screened for the greatest antitumor activity and rapidly expanded before reinfusion. The ability of TILs to induce tumor regression was enhanced by administering chemotherapy to patients prior to TIL infusion, which eliminated immune cells that mediate tolerance, while increasing the levels of homeostatic cytokines IL-15, IL-17, and possibly IL-21 (74–76). Further, components of anti-tumor immunity in the TME, such as myeloid-derived suppressor cells and T regs, were shown to be diminished following this procedure (77–79). In one study, patients with early stage NSCLC either received chemotherapy with ACT or chemotherapy alone following surgical resection of their tumors (80). Patients in the chemotherapy-ACT group had better disease-free survival rates (80). More studies employing ACT for cancer treatment are currently underway (NCT02998528).

### Adoptively Transferred Tumor Reactive T Cells

Despite its promise, the success of ACT was primarily restricted to melanoma patients, which was later attributed to the disease's inherent immunogenomic landscape; melanomas exhibit high mutation rates which produce tumor-specific antigens, or neoantigens, highly recognizable by TILs (81). This has prompted the investigation of adoptively transferring TILs that were specifically tumor-reactive, that is, genetically engineered to respond to tumor-specific mutational epitopes (82). Peripheral T cells were then engineered to express TCRs against specific tumor neo-antigens in highly mutated tumors such as melanoma and lung cancer (83–90). Adoptively transferred genetically engineered lymphocytes directed against specific NY-ESO-1 epitopes, a well–known cancer-testis antigen, have shown promising clinical responses across a number of tumors (35). This and other potential TCR targets are currently being investigated, as part of engineered T cell treatments, in clinical trials for lung cancers including NSCLC (NCT03778814) (35).

In depth investigation of the mechanism of action of TCR-engineered TILs identified the need for MHC recognition as a major limitation due to the MHC-downregulating nature of some tumors. To circumvent that, co-transduction of a chimeric antigen receptor (CAR) was investigated (36). CARs are synthetic receptors that enhance T-cell antitumor effector function and produce superior anti-cancer activity as seen in successful outcomes of clinical trials treating patients with B cell hematologic malignancies (91). The first clinical application of CAR-T cell therapy involved creating a receptor that recognizes the CD19 antigen in the treatment of a patient with follicular lymphoma refractory to treatment (17). Dramatic tumor regression was observed, which drove the investigation of CARs in other malignancies and solid tumors. However, the complex nature of the solid TME has restricted the efficacy of CAR-T cell therapy and posed challenges in finding ideal target antigens that are highly and selectively expressed on cancer cells, with minimal to no expression on normal counterparts. Albeit with a handful of potential promising antigens (such as MAGE, mesothelin, NY-ESO-1, ROR1, WT1), CAR-T cell therapy is being investigated in phase I and phase II clinical trials in a number of solid malignancies, including LUADs, LUSCs, and mesothelioma (91).

Furthermore, natural killer (NK) cells have been recently catching up as the new CAR-engineered cells besides TILs. The advantages that CAR-NK cells possess over CAR-T cells have been quite evident in several pre-clinical reports, and those include: reduced risk of overexpansion in patients, release of safer cytokines thereby reducing/eliminating the risk of life-threatening cytokine release syndrome (CRS)–the most common and severe side effect of CAR-T therapy, and eliminating the need for tumor-specific surface receptors and autologous production which potentiates the concept of “one CAR-NK fits all” (18). Additionally, one study reported, in mice, an anti-tumor effect of CAR-NK cells differentiated from induced pluripotent stem cells *in vitro*, with significantly reduced adverse effects compared to CAR-T cell therapy (19). While the field remains in its infancy, CAR-NK cell therapy is now being clinically tested against a handful of hematological malignancies (NCT02742727) with new trials to be soon launched, while more pre-clinical studies are warranted to validate its efficacy in other malignancies including solid tumors.

### Cancer Vaccines

Cancer vaccines constitute an immunotherapy strategy that is designed to target tumor-specific or tumor-associated entities, encouraging the microenvironment to attack them by boosting T cell, or B cell-mediated antitumor response. Tumor cell vaccines could be either autologous, that is produced from the patient's tumor cells, or allogeneic, that is derived from human tumor cell lines. Depending on the target entity, these vaccines are classified into different categories such as cell-based vaccines (whole tumor vaccines), genetic vaccines (DNA vaccines), protein vaccines, bacteria vaccines, small molecule vaccines, and DC-based vaccines (25).

The concept of cancer vaccines was initially proposed by Coley who introduced inactive forms of the bacteria *Streptococcus pyogenes* and *Serratia marcescen*s into sarcoma patients *via* intratumoral injections. The bacteria elicited an inflammatory reaction causing the sarcomas to shrink in size (92). Later, tumor vaccines comprised of melanoma–associated antigen-A3 (MAGE-A3) were used as an adjuvant to surgery in the treatment of early stage MAGE-A3+ NSCLC in a large scale phase III clinical trial. The study found no survival benefit in patients who received the vaccine in comparison to those who received a placebo (27). Another evaluated vaccine was Tecemotide, which is an analog of mucin-1, a glycoprotein found to be overexpressed in NSCLC (28). Patients receiving Tecemotide tumor vaccine following treatment with chemoradiation had no survival benefit compared to controls (28). Despite breakthrough results in pancreatic cancer patients for instance (29), the efficacy of cancer vaccines in the treatment of pulmonary malignancies remains controversial. However, several other tumor vaccines are currently being evaluated in clinical trials for lung tumors, and those target entities (mostly antigens or proteins) that are specific or highly abundant in lung cancer, such as 5T4, CEA, mesothelin, survivin, NY-ESO-1, telomerase, WT1, EGFR pathway proteins, in addition to personalized neoantigens and tumor-associated antigens (TAA) (which are further described below) (30, 93, 94).

### Oncolytic Viruses

Oncolytic viruses (OV) are DNA or RNA viruses that have the ability to replicate and kill cancer cells in a targeted and specific manner, particularly since they lack virulence against non-malignant cells (11, 95). Oncolytic virotherapy has been tested in pre-clinical and clinical settings by intratumoral administration of viral particles delivered locally to the tumor (96). Cancer cells infected with OVs self-destruct, thereby attracting the attention of immune cells such as TILs or neutrophils leading to the production of inflammatory mediators, consequently eliminating the main tumor and potentially other tumors within the body. OVs can also induce viral infection cascades known to elicit a type 1 interferon response thereby stimulating cytokine release, cancer cell lysis, and apoptosis (97, 98). The first OV approved by the FDA was Imlygic, a herpes simplex virus I tweaked to preferentially kill cancer cells, which was approved in 2015 for the treatment of advanced melanoma (39, 99). TG4010, an attenuated poxvirus engineered to express MUC-1 and IL-2 prolonged the survival of patients with advanced NSCLC when administered with first-line chemotherapy and is now being tested in phase III clinical trials (43). Interestingly, TG4010 showed synergistic effects when used in combination with anti-PD-1/PD-L1 ICB in murine models (44). Other OVs being evaluated in lung cancer clinical trials include viruses derived from adenovirus, picornavirus, reovirus, as well as coxsackie, herpes simplex, maraba, measles, and vaccinia virus (39, 100, 101).

### Targeted Antibodies

Targeted antibodies are antibodies customized to recognize specific cancer cell antigens. In addition to the use of cancer-specific monoclonal antibodies (mAb), two potent customizations, namely antibody-drug conjugates (ADCs), and bi-specific T cell-engaging antibodies (BiTEs) are being tested as anti-cancer immunotherapies, several of which have been approved primarily for hematological malignancies (45, 47). ADCs are highly potent constructs of tumor-specific mAbs equipped with anti-cancer drugs which are effective once internalized by a tumor cell (45). BiTEs provide enhanced efficacy and safety by simultaneously binding a cancer cell antigen and the CD3 of T cells, thereby directing the host immunity toward a T cell–driven cytotoxic antitumor immune response (47). Antibody targets under evaluation in lung cancer clinical trials include: cMET, DLL/Notch, FGF/FGF-R, HER2, mesothelin, PDGFR-alpha, TROP2, and lastly EGFR and VEGF/VEGF-R, whose respective targeting antibodies necitumumab and bevacizumab, have been approved for subsets of patients with advanced NSCLC, including as a first-line therapy (102–106).

### Immune Checkpoint Blockers

Currently, ICBs are being tested in advanced stages of clinical trials (107, 108). So far, six immune checkpoint blockers (ICBs) have been FDA-approved for the treatment of liquid malignancies (Hodgkin's lymphoma) as well as solid tumors such as lung, skin, head-and-neck, and bladder cancers (108–112). Two of the most widely studied ICBs comprise mAbs that block PD-1 or CTLA-4 (62), both of which inhibit proteins that halt the immune system, thereby reinvigorating a robust endogenous antitumor immunity.

As described above, CTLA-4 was identified as the prototypical negative regulator of T cell activation, which was seminal in understanding that T cell activation by “signal one” can be achieved by removing inhibitory molecules in the co-stimulatory pathway. This provided rationale for blocking CTLA-4, thereby enabling the induction of a positive co-stimulatory signal through CD28/CD80 engagement and re-activation of “signal two” whereby T cells proliferate and effector cytokines such as IFN-γ are produced (113). The proof-of-concept came from results in animal experiments showing tumor regression 30 days after treatment (114, 115). Fourteen years later, a large multicentric trial showed improved survival for ipilimumab, a fully humanized mAb against CTLA-4, in metastatic melanoma patients (116). This response was a turning point in cancer immunotherapy clinical trials, and prompted the approval of ipilimumab by the FDA in the following year, making it the first approved ICB for cancer treatment. Later studies showed that anti-CTLA-4 treatment induces a robust increase in both CD4+ and CD8+ T cell populations in several human cancers including lung cancer, thereby limiting T reg infiltration into the tumor bed and increasing T effector to T reg ratio (51, 55, 59, 66, 117) (Figure 1). Clinical trials in NSCLC and SCLC patients showed little promise for anti-CTLA-4 treatment in combination with chemotherapy, although current studies are exploring combinations with other ICBs with non-overlapping functions, such as anti-PDL1/PD-1 mAbs, further described below.

In the early 2000s, and decades after its discovery and cloning, PD-1, along with its ligand PD-L1, was identified as a regulator of T cell activation and immune exhaustion (118). In peripheral tissues, PD-1 –expressing T cells interact with immunosuppressive PD-1 ligands PD-L1 (B7-H1) and PD-L2 (B7-DC) found on tumor cells, stromal cells, or both (119–122). Blocking this interaction with anti-PD-1/PD-L1 antibodies enhanced T cell responses *in vitro* and mediated preclinical antitumor activity (119, 123) (Figure 1). In a phase I clinical trial of PD-1 mAb, dramatic anti-tumor responses were reported among 236 treated patients with various types of cancer, including 18% of patients with advanced NSCLC patients (124). Additionally, tumors with high PD-L1 expression, defined as at least 50% of expression specific to tumor cells, exhibited improved response to anti-PD-1 monotherapy in NSCLC patients (125). This was further confirmed in a landmark study involving metastatic NSCLC treatment-naïve patients who were allocated to two treatment groups, chemotherapy or pembrolizumab, a highly selective humanized PD-1 mAb. Patients expressing at least 50% of PD-L1 on tumor cells achieved superior progression-free survival with anti-PD-1 compared to chemotherapy, with a durable overall survival of 60.6% for 24 months (126). Also, 73.4% of those patients exhibited adverse side effects in comparison to 90% of the patients receiving chemotherapy (126). This study also confirmed that 23–28% of NSCLC patients express at least 50% of PD-L1 on tumor cells, therefore unleashing the potential antitumor ability of inhibiting this checkpoint axis in NSCLC (126). In 2019, the FDA approved pembrolizumab as first-line treatment of patients with stage III NSCLC that is PD-L1-positive and not amenable to surgery or chemo-radiation treatment (9, 127). In addition, metastatic SCLC patients whose tumors progressed after treatment with platinum-containing chemotherapy and at least one other systemic therapy, were approved for nivolumab treatment in 2018, an anti-PD-1 ICB, as the first immunotherapy approved for SCLC (128). Other PD-1/PD-L1 pathway-targeting ICBs approved for specific subsets of lung cancer patients include atezolizumab (NSCLC and SCLC patients) including as a first-line therapy, durvalumab (advanced NSCLC), and nivolumab (advanced NSCLC and a subset of metastatic SCLC as described above) (49, 129–131).

Later studies showed that clinical efficacy of anti-PD-1 treatment was also achieved in a subset of lung cancer patients, among which 15–40% harbored low or no tumor-specific PD-L1 expression (125). In fact, targeting the PD-1/PD-L1 axis showed variable levels of efficacy across multiple tumor types and among patients with the same type of cancer, due to several factors such as gender, driver mutations, genomic instability (such as translocations in *ALK, KRAS, EGFRI* in lung cancer patients), and the degree of tumor metastases (62, 132).

An additional robust predictive biomarker for anti-PD-1 efficacy in lung cancer patients was therefore needed, and this stemmed from our understanding of the successes of checkpoint blockade in melanomas. In melanomas, high tumor mutational burden (TMB) is a critical prognostic marker for anti-CTLA-4 treatment (133). This has been extended to NSCLC patients receiving anti-PD-1 therapy, particularly since lung carcinomas are the second top human malignancies with the highest TMB, after skin-related malignancies (133, 134). A seminal study confirmed an objective response rate of 19.4% and a median overall survival of 12 months following pembrolizumab treatment in patients with advanced NSCLC (135). Current or former smokers achieved a response rate of 22.5%, while never-smokers had a response rate of 10.3% (135). This was attributed to increased carcinogen-induced TMB which correlates with better ICB efficacy. Indeed, a smoker molecular signature correlates with higher prevalence of somatic non-synonymous point mutations, an increased TMB, and elevated neoantigen production (133). Treating such immunogenic tumors with anti-PD-1 subsequently increased T cell clonality which drove an antitumor immune response (136). Indeed, 73% of patients with considerably high TMB exhibited durable clinical efficacy of pembrolizumab (136). Patients exhibiting durable clinical benefit showed a median of 302 non-synonymous mutations, compared to 148 in those with no clinical benefit (137). Further, tumors of never-smokers generally harbored fewer somatic alterations and displayed reduced anti-tumor response to PD-1 blockers, thus accentuating the role of neoantigen-specific effector T cell responses in enhancing tumor recognition and attack (137) (Figure 1). Based on the combined data from disease-specific pembrolizumab clinical trials (KEYNOTE-016, KEYNOTE-164, KEYNOTE-012, KEYNOTE-028, and KEYNOTE-158), 2017 witnessed the accelerated and first ever biomarker-based, as opposed to organ-specific tumor type, FDA approval of pembrolizumab as a second-line treatment for all metastatic solid tumor types classified as microsatellite instability (MSI)-high or with deficient DNA mismatch repair (138, 139). Building on this, any failure to deliver survival benefits in PD-L1-high/TMB-high/MSI-high patients receiving ICB therapies could be attributed to insufficient CD8+ T cells infiltrating the tumor bed, hypoxia, mutation variability in oncogenic pathways, intratumoral heterogeneity of cytotoxic T cells populations, or specific human leukocyte antigen (HLA)-restricted neoantigens (62) (Figure 1). This also highlighted a window of opportunity to treat early stage tumors with immune checkpoint therapies, a time point at which the immune landscape, characterized by T cell exhaustion, is still reversible and can thus be targeted by ICB (140).

### Combination ICB

The majority of patients treated with single-agent ICB exhibited promising durable disease control, yet, oftentimes their insufficient capability to activate an antitumor immune response resulted in patient relapse and tumor resistance to ICB (54, 141). Therefore, the potential of combining two or more immunotherapies in hopes of achieving synergistic effects became an intriguing prospect, with documented survival advantages in comparison to monotherapy in certain cancer types (66, 108, 142–144). The focus of clinical trials was now shifting from sequential monotherapies to evidence-based examinations of the potential of combining multiple therapies with non-redundant anti-tumor activities. Indeed, the two best-studied ICBs, anti-CTLA-4 and anti-PD-1 mAbs, mediate distinct yet complementary antitumor responses despite both having a suppressive effect on T cells (55, 145–147). For instance, CTLA-4 modulates the proliferation of T cells by blocking auto-reactive T cells, primarily in lymph nodes, and at the early priming stages of immune activation. On the other hand, PD-1 suppresses T cell activation at the later effector phase and in peripheral sites, and it is expressed on a broader variety of cell types in comparison to CTLA-4 (55, 66, 145–148). The complementarity of the blockers' mechanism of action was the rationale for investigating dual ICB.

Nivolumab along with ipilimumab concurrent therapy for late stage melanoma patients achieved clinical success, including improved objective response rate, progression free survival, a stable improvement in survival benefit, and reduced toxicity in comparison to patients receiving nivolumab alone (134, 142, 149–151). Improved response to dual ICB seemed to override the need for abundant PD-L1 expression observed in monotherapy, whilst high TMB and improved response correlated well-across the dual ICB group (134). It was also reported that combination immunotherapies could not deliver survival benefit among NSCLC patients with low TMB, which is consistent with data from gastrointestinal cancers (134, 152). While some combinations were showing reduced toxicities compared to single-agents, other immune-related adverse effects were being reported, which may be reversed if properly addressed (153, 154). The main advantage that combination therapy provides lies in the enhancement of TIL, which subsequently leads to increase in effector cytokines that shift the tumor microenvironment toward an immuno-active milieu (143, 155). In addition, dual ICB was clinically beneficial in another group of advanced melanoma patients, whereby the combination lead to intracranial and extracranial activity in asymptomatic untreated brain metastases in 57% of the patients (156, 157). Combination ICB is proving to be a promising avenue worth further investigation, particularly since it has the potential to turn immunologically cold tumors into hot tumors, as seen with prostate tumors which are known to be resistant to PD-1 blockade due to their lack of TILs. In one cohort, anti-CTLA-4 increased expression of PD-1 and VISTA inhibitory checkpoints in prostate tumors, which makes targeting CTLA-4, PD-1, and VISTA an appealing combinatorial approach (158).

To date, the FDA has approved 20 single-agent and only three combination immunotherapies (159–161). Although anti-PD-1 and anti-CTLA-4 lie at the heart of monotherapies, other “next-generation” molecules are now being considered particularly as co-targets for combination immunotherapies. While blocking CTLA-4 and PD-1 could occlude overall immune self-tolerance, it is proposed that co-targeting novel molecules such as TIGIT, LAG-3, and TIM-3 would exert more specific roles, which could improve safety profiles (53). Indeed, combining checkpoint and non-checkpoint blockade-based immunotherapies may render cold ICB-resistant tumors sensitive. In a preclinical model, mice receiving intratumoral injections of toll-like receptor 7 (TLR-7) agonists accompanied by anti-PD-1 had a better chance of activating APCs, due to TLRs' potential in inducing robust antitumor activities by affecting both the innate and adaptive immunity (162, 163). This also induced a dramatic increase in CD8+ T cell clones (163). Additional approaches involve ICB combined with other immunotherapies (ACT, OVs, cancer vaccines, mAbs), epigenetic modifiers, targeted therapies, or conventional therapies (e.g., radiotherapy and chemotherapy, such as FDA-approved atezolizumab and chemotherapy for SCLC) (164–167).

## Immunogenomics in Lung Cancer: Behind the Scenes of the Mechanisms of Immunotherapies

As outlined above, there have been great strides in our understanding of which cancers are likely to respond positively to certain immunotherapies, mostly based on knowledge of the pre-existing genomic landscape of tumors that is pertinent to the immune environment. However, the picture remained far from complete, and this can be deducted from recurring observations across several clinical trials, including: resistance to some immunotherapies, adverse tumor progression, toxicity and side effects, among others (168–172). A step back to understanding the immune biology of naïve tumors was thus crucial, one that delves into modern genomic and proteomic tools to decipher the interaction of a tumor cell with its microenvironment in a tumor-type specific manner, that being at the genomic resolution rather than merely the pathological one. Immunogenomics was then brought into perspective, as the go–to approach that holds the key to bringing immunotherapy to its best possible potential. This was possible with the advent of next generation tools to assess tumors and their microenvironment, such as next-generation sequencing, single cell RNA sequencing, mass cytometry by time-of-flight, immune cell profiling (T cell and B cell receptor sequencing), as well as RNA sequencing of the 16S ribosomal subunit specific to host microbial species.

### Tumor Neoantigens

As previously mentioned, studies have repeatedly shown that tumors with a high mutational load possess a high number of neoantigens and are more responsive to ICB (173). ICB results in the stimulation of a plethora of neoantigen-specific cytotoxic T lymphocytes that attack the tumor resulting in remission (174). Tumors with a high mutational burden like NSCLC are more responsive to ICB than tumors with a low mutational load like clear-cell renal cell carcinoma and breast tumors (136, 137, 175). This makes ICB a more logical approach for tumors with a high mutational load, and tumors with a low mutational load may best be addressed with more conventional forms of cancer treatment or other forms of immunotherapy.

Yet, some tumors with high mutational load are surprisingly unresponsive to ICB treatment, and a deeper investigation revealed additional factors that influence neoantigen presentation and response to ICB. For instance, it has been proposed that the quality of neoantigens provide a better reflection of tumor immune landscape compared to the quantity of neoantigens and this has been possible thanks to neoantigen prediction algorithms and advanced *in silico* models (176–179). For instance, long-term survival of patients with pancreatic cancer correlated with neoantigen “foreignness,” an attribute of their homology to antigens derived from infectious diseases, rather than the actual number of neoantigens, a timely observation given the emerging roles of tumor microbiomes (further discussed below) (177). The relationship between neoantigen burden and response to immunotherapy is further controlled by their genetic heterogeneity within a single tumor, or neoantigen intratumor heterogeneity. Neoantigen clones can be either present on all tumor cells (clonal), or only a fraction of cells (subclonal), and it is the clonal neoantigens that are specifically correlated with improved patient survival, an increased presence of TILs, and a durable response to immunotherapy among melanoma and lung cancer patients (87). Thus, a high burden of neoantigens *per se* does not necessitate effective antitumor immunity in response to immunotherapy. Interestingly, smoking-associated NSCLCs have an extensive clonal mutational repertoire, and subclonal tumors are more likely to acquire resistance to ICB (180–182). Therefore, a deeper understanding of the mechanisms causing subclonality, which arises from chromosomal deletion of the genes coding for the targeted neoantigens or by elimination of all tumor cells presenting the neoantigen, is warranted (182).

### HLA Haplotypes

Another factor modulating response to ICB and overall immune-evasion is the patient's HLA. The role of neoantigens as critical players in tumor immune-evasion and response to immunotherapy is governed by presentation on HLAs to be recognized by T cell receptors. Therefore, losing the ability to present neoantigens can be further mediated by HLA (or MHC) molecules themselves and may thus modulate tumor immunity (Figure 1). In a landmark study, researchers studied the association between HLA-genotype and 1,018 oncogenic mutations in 9,176 cancer patients (183). Some mutations were disproportionately associated with certain HLA genotypes but not others. Incidentally these mutations were poorly presented on the tumor (183). The results make sense if interpreted in the context of the immunosurveillance theory (71). The HLA genotype influences which mutations will be represented as neoantigens which are subsequently eliminated by the immune system in the elimination phase. Mutations that survive this phase evade immune surveillance by being poorly presented on HLA molecules and are thus more likely be found in the tumor later on. Indeed, some tumors were shown to downregulate MHC-1 expression on which neoantigens are presented (184). Tumors with lower MHC-1 expression have poorer prognosis, and this has been documented in multiple tumor types including NSCLC (185). NSCLCs with decreased MHC expression also had decreased T cell infiltration and a more immunosuppressive microenvironment (185). The decrease in expression was accomplished by either HLA loss of heterozygosity (LOH) or downregulation of β2-microglobulin, which is a crucial component of MHC (185). A study by McGranahan and colleagues employed immunogenomics tools to identify HLA-specific copy number from sequencing data, from which they were able to derive and characterize HLA LOH patterns among early stage NSCLCs (186). They identified that 40% of early stage NSCLCs harbor HLA LOH and that this is significantly associated with a high subclonal neoantigen burden, APOBEC-mediated mutagenesis, upregulation of cytolytic activity, and PD-L1 positivity (186). Further investigation suggested that HLA LOH is an immune escape mechanism that is subject to strong selection pressures from the microenvironment thereby influencing tumor evolution. Additionally, the affinity with which HLAs bind to their respective neoantigens has been a subject of interest in cancer immunogenomics, and an area that is expanding *in silico* immunogenomic tools, such as neoantigen prediction software which are being used to predict both HLA binding affinities for each tumor-specific antigen (179, 187).

### Somatic Mutations

Somatic mutations generate neoantigens and may also influence response to ICB in a gene-specific manner, and next generation sequencing has been instrumental in identifying the somatic alterations that influence the immunogenomic landscape of tumors (see Table 1). In a recent study, researchers established that *KRAS*-mutant LUADs harboring additional mutations in *STK11/LKB1* were unaffected by PD-1/PD-L1 inhibition (210). Indeed, *KRAS* is one of the most commonly mutated oncogenes in LUADs, prevalent in 33% of LUAD cases (4). LUAD patients harboring *STK11* mutations were shown to have low densities of CD8+ TILs in tumor beds, in contrast to *STK11* wildtype LUAD patients who displayed high levels of CD4+ and CD8+ T cells (211). Mechanistically, inactivating mutations in the tumor suppressor *STK11* were able to reprogram the TME into so-called “immune-deserts,” which support a “cold” tumor (184, 210, 212). When treated with anti-PD-1 therapy, *KRAS*-mutant LUAD tumors with co-occurring mutations in *STK11* had an objective response rate of 7.4%, compared to the highly responsive subgroup of *KRAS*-mutant LUADs with *p53* mutations showing 35.7% objective response rate (210). Additionally, *STK11* was identified in this study as the sole genomic alteration that is significantly enriched in PD-L1 negative tumors with an intermediate TMB profile (210). This study asserts that *STK11*-mutant LUAD tumors generally lack PD-L1 expression and thus correlate with minimal disease control post-ICB therapy regardless of TMB status (210). Hence this genomic driver mutation could be a major driver for *de novo* resistance to PD-1 axis blockade in LUAD.

**Table 1:** Changes in tumor immune microenvironment of preneoplastic lesions and lung cancer.

**Pan lung cancer preneoplasia**	**Lung cancer preneoplasia**	**Lung cancer**
↓ Th1-dervied IFN-γ ↑ Th2 in Barret esophageal tissue ↑ Pro-inflammatory mediators (*IL-17A, IFN-γ, IL-6*) in OPLs (188, 189)	↓ Anti-tumor Th1 (*IL12A, GZMB*) ↑ Pro-tumorigenic Th2 ↑ Pro-inflammatory cytokines (*IL-6, TNF-α*) in AAH (190, 191)	↓ Anti-tumor Th1 (*IL12A*) ↑ Pro-tumorigenic Th2 ↑ Immune suppressive mechanisms (*IL6, IL10*) (191, 192)
↑ Immune checkpoints (PD-L2, LAG-3) in Lynch syndrome (193)	↑ Immune checkpoints (CTLA-4, CCR2) (191)	↑ Immune checkpoints (PD-1, CTLA−4, VISTA, LAG−3, TIM-3) (191)
↑ CD4+ and CD8+ TILs (194)	↑ Exhausted CD8+ TILs reactive to neoantigens (195)	↓ Cell–mediated immune response (195)
Uncontrolled TLR signaling (196, 197)	↑ TLR and inflammatory mediators (NF–κB) ↑ downstream chemokines (IL-6, IL−17) (198)	↓ TLR, ↓ Effector cytokine production (IFN–γ, TNF–α) (199)
Progressive infiltration of innate immunosuppressive cells and M2 macrophages and T regs in OPLs (188, 200)	Immature macrophage-lineage cell infiltration (201)	Massive tumor immune cell infiltration (201)
↑ B-cell chemotaxis (↑ CXCL13, CXCL14) (202)	↑ B-cell chemotaxis (↑ CXCL13, CXCL14) (191)	↑ B-cell chemotaxis (↑ CXCL13, CXCL14) (191)
Common tumor antigens between cancers and PMLs (203)	↑ Neoantigen expression in due to infiltration of CD4+ and CD8+ T cell as well as ↑ PD-1 (179)	↑ Immunogenic neoantigen load activating anti-tumor T cell response (195)
Humoral cell-mediated immune response activated against TAA in gastric premalignant lesions (204)	Activation of cell-mediated immune response and recognition of neoepitopes (179)	Activation of cell-mediated immune response and recognition of neoepitopes (179)
Very few chromosomal mutations (*TP53* in Barret's esophagus) (205)	↓ Somatic mutational processes (*TP53*) (205)	↑ Tumor mutational landscape (*KRAS, BRAF, EGFR, TP53*) (205)
AI in oropharyngeal epithelial dysplastic lesions (LOH and MSI) (206)	Genome-wide spatial gradient of AI next to tumor sites (205)	Genome-wide spatial gradient of AI next to tumor sites (205)
LOH in chromosomal arms 3p, 17p, 13q in OPLs (207)	LOH in chromosomal arms: 17p, 13q, 19pl, and 9q (191)	LOH in chromosomal arms: 17p, 13q, 19p, and 9q (191)
Epigenetic changes in oral PML (*TP53, CDKN2A, PIK3CA, HRAS)* (208)	Epigenetic modifications (*CDKN2A*) and differential gene expression patterns in AAHs (205)	Epigenetic modifications (*CDKN2A*) and differential gene expression patterns in AAHs (209)

*OPL, oral premalignant lesions; TILs, tumor infiltrating lymphocytes; TLR, Toll-like receptor; T reg, regulatory T cell; PML, premalignant lesion; TAA, tumor-associated antigen; LOH, loss of heterozygosity; MSI, microsatellite instability; AI, allelic imbalance; AAH, atypical adenomatous hyperplasia*.

### TILs

Evaluation of TILs provides an estimate of the abundance of certain immune subsets which can be of prognostic value particularly when such correlates are evident at early stages of tumors, as seen in the case of lung cancer. Additionally, the repertoire of immune receptors, including immune checkpoints, found on TILs was shown to be an important regulator of maintenance of a tumor-reactive state for TILs, which improves the outcome of ICB in cancer patients. The ability of certain tumors to warp TIL landscape to a cold, immunosuppressed, hypofunctional state has been shown to be regulated at early stages of tumorigenesis. This has been further demonstrated in a mechanism of TIL exhaustion in NSCLC, whereby early on, TIL function can be regulated by a competition between anti-tumoral and pro-tumoral/exhaustive events elicited by tissue-resident memory cells (213).

### Microbiome and Tumor Immunity

While emerging technologies such as next generation sequencing have allowed an unprecedented understanding of the genomic landscape of tumors and their TME including immune cells, it has also extended to encompass characterization of other influential components of the tumor milieu. These emerging players are the microorganisms that naturally inhabit various niches in the host, including the respiratory system and the alimentary tract, or the microbiota. As such, the role of the genomic architecture of these organisms, termed *microbiome*, as a new frontier in human and animal medicine, has become a standard and expanded dramatically in the past decade (214). Microbiota play a central role in the induction, differentiation, and overall function of the immune system (215). It maintains immune homeostasis by influencing the differentiation and production of anti-inflammatory cells and cytokines including T regs, tolerogenic DCs, IL-23, IL-33, and TGF-β (216). It has become evident that intact and balanced microbiota, on its own merit, has a positive impact on the health and physiology of the host by influencing a variety of biological functions ranging from behavior, to obesity and cancer (217). Indeed, the microbiome has been dubbed as a “key orchestrator of cancer therapy,” modulating chemotherapy, radiotherapy and immunotherapy (214). Earlier work demonstrated that the host (e.g., gut) microbiome dynamically interacts, i.e., influences and is affected by, inflammation including pro-tumor inflammatory pathways, and that microbial dysbiosis is implicated in many tumors, notably lung carcinomas (218, 219). Of note, preclinical studies have identified gut bacteria that influence tumor immunity and response to immunotherapy and chemotherapy, in addition to other seminal reports whose findings accentuate the role of the host microbiome in tumor biology, as discussed below (220–222).

In a recent study, researchers assessed and clustered the fecal microbiome composition of 26 patients with metastatic melanoma prior to treatment with ipilimumab (223). Patients whose fecal microbiota was enriched with the *Faecalibacterium* genus and other *Firmicutes* had improved responses as indicated by longer progression free survival and longer overall survival compared to those whose microbiota was enriched with *Bacteroides* (223). In addition, these patients exhibited a significantly lower proportion of T regs, α4+β7+ CD4+, and α4+β7+ CD8+ T cells compared to patients from the *Bacteroides* cluster (223).

Microbiome composition also influences the response to PD-1/PD-L1 checkpoint inhibitors. By investigating the influence of oral and gut microbiome on response to anti-PD-1 immunotherapy in metastatic melanoma patients, researchers found that the diversity, and composition of the oral microbiome had no effect on treatment outcome (224). However, responders had higher α-diversity and relative abundance of *Clostridiales/Ruminococcaceae* as compared to non-responders who had lower α-diversity and abundance of *Bacteroidales* (224). Moreover, the *Clostridiales/Ruminococcaceae*-enriched cohort had a higher fraction of TILs, circulating CD4+ and CD8+ lymphocytes, overall density of immune cells, and increased expression of markers of antigen processing in the myeloid compartment (224). In contrast, patients whose oral and gut microbiota were enriched with *Bacteroidales* had a higher percentage of circulating T regs and myeloid derived suppressor cells (224). These results provide insight into mechanisms by which the microbiome influences the response to checkpoint therapy. In a similar study, *Akkermansia muciniphila* was found to be abundant in the intestinal microbiome of patients with NSCLC responding to PD-1 blockade (225). The authors noted that *A. muciniphila* increases the recruitment of CCR9+CXCR3+CD4+ T lymphocytes to tumor beds in an IL-12-dependent manner (225). These cells may increase the number of TILs by secreting chemokines that induce migration (184).

This impact of the microbiome on tumor immunity extends beyond landscaping a positive response to ICB to further mitigate one of the most frequently described side effects to ICB, enterocolitis (226). This adverse condition usually presents as a triad of diarrhea, abdominal pain, and vomiting (227). Enterocolitis is particularly frequent and severe following CTLA-4 inhibition, and appears to resemble, and even cause inflammatory bowel diseases (228, 229). In the above-mentioned report utilizing anti-CTLA-4 antibodies, *Faecalibacterium* genus and other *Firmicutes* not only correlated with a good treatment outcome, but also with the frequency and severity of enterocolitis (223). In another study, researchers identified *Bacteroides* species as a protective factor against the development of enterocolitis (230).

While most studies focused on the modulatory effects of the microbiome in response to immunotherapy, there have been some promising attempts to explore a potentially prominent relationship between the microbiome, immune system, and cancer in the preventive setting. In animal models, modulation of the lung microbiome using probiotics or antibiotics decreased tumor seeding in the lung and improved the effect of chemotherapy against experimental metastases (231). This was accompanied by antibiotic- or probiotic-mediated reduction of immunosuppressive cells in the lung and maturation of resident antigen presenting cells, respectively, (231). Fecal microbiome transplants from long-term pancreatic cancer survivors were also shown to modulate tumor immunosuppression and growth in mice, thereby providing rationale for microbiome interference to target ICB-refractory pancreatic tumors presumed to be poorly immunogenic (232). Further investigations lend evidence to the possibility that early microbiome imbalance may dysregulate host immunity leading to the immune escape of premalignant lesions, warranting preclinical as well as clinical validations with the potential of deriving non-invasive microbiome-based biomarkers for early detection of cancers (233, 234). In the context of lung tumors, studying the microbiome can be a low-hanging fruit for the derivation of early detection markers for several underlying reasons. First, activation of pro-tumor inflammatory pathways, an enabling hallmark of cancers, reprograms the tumor immune microenvironment to promote lung carcinogenesis (9, 201, 235). This is initiated by intrinsic (e.g., oncogene activation) or extrinsic (e.g., infection or smoke-induced) mechanisms. For instance, exposure to tobacco smoke can disrupt lung epithelium, thereby reducing microbiome diversity and promoting pathogenic bacteria to dominate, all while making the normally commensal species more virulent (236). In a vicious cycle, this could further increase epithelial inflammation and predisposition to malignant transformation. Future studies ought to characterize tumor immunogenomics at the level of the microbiome too, particularly in early stages of carcinogenesis. In one report, LUSCs with somatic mutations affecting *TP53*, the most commonly mutated tumor suppressor gene in lung cancers, were enriched with tissue-specific microbial consortia, a subset of which was highly abundant among smokers (237). Thus, in addition to known gene-environment interactions, further investigation into gene-microbiome interactions is warranted, as they seem to be evident early on during lung carcinogenesis, providing an environment conducive to particular microbial species, and possibly acting as promoters of tumorigenesis.

## Rewinding Lung Carcinogenesis: The Antecedents of Cancer Invasion, and an Opportunity for Early Intervention and Immune Prevention

There is an urgent and unmet need for new strategies for early detection and management of the most common killer among all cancers (238, 239). Limiting these advances is a poor understanding of the earliest events that drive lung cancer development and that would thus be ideal targets for early treatment. It is worthwhile to mention that there are few molecular alterations that have been described in premalignant phases of lung cancers, particularly LUADs and LUSCs. However, while physical screening, identification and thus molecular characterization of the earliest premalignant lesions (PMLs) remains hampered by their elusive pathology, understanding their immune biology is gaining momentum as a promising surrogate for the early detection of lung cancers, and as a window of opportunity to intercept their progression using novel immune-based prevention strategies (240). Indeed, successful interventions at premalignant stages were evident across multiple tumors. For instance, oral premalignant leukoplakias (OPLs), the PMLs and precursors of oral squamous cell carcinoma, were found to harbor increased expression of the immunoinhibitory mediators HLA-G, -E, PD-L1, IL-10, TGF-β2, and -β3 relative to the normal tissue, and with levels resembling those analyzed in oral squamous cell carcinoma tissues (241). A murine carcinogen-induced premalignant lesion model that progresses to invasive disease revealed dynamic shifts in OPL immune environment with disease progression, and this was characterized by an increase in OPL inflammatory mediators and IL-17 that declines with disease progression (188). Further, PD-1 blockade in a carcinogen-induced leukoplakia mouse model reduced the number of OPLs and their frequency of transforming to oral squamous cell carcinomas (242). The treated OPLs displayed increased recruitment of CD4+ and CD8+ T cells with an accompanying increase in IFN-γ, granzyme B, and STAT1, as well as an increased recruitment of CTLA-4 positive cells, suggesting that a combination of CTLA-4 and PD-1 blockade may have synergistic effects in the treatment of OPLs (242). A deeper understanding of cancer immunogenomics at the earliest stages of cancer and PMLs has led to medical breakthroughs in screening, early detection and prevention, as seen with the introduction of HPV vaccines and monitoring of Barrett's esophagus (243, 244). We envision a similar approach in future investigations of lung cancer premalignancy, which has accumulated mounting genomic evidence supporting its critical roles in the switch to lesion progression. We also surmise that a particular focus on lung PML immune biology will complement the current prevention methods, and introduce new immune-based interceptive therapies (see Table 1). Indeed, with the advancement in immunogenomics profiling techniques, probing the role of immunotherapy in early stage lung cancer has expanded, starting with its characterization in early malignancy, to neoadjuvant/adjuvant intervention, as well as in animal models, chronic obstructive pulmonary disease (COPD) cohorts, and in unique patient cohorts with detectable PMLs.

### Immuno-Adjuvant and Neoadjuvant Therapy in Early Stage Lung Cancer

In the past decade, and while exploration of the immune landscape of PMLs was still in its infancy, immunotherapy was tested in early stage lung tumors with promising results (245, 246). Interestingly, early-stage LUADs treated with standard adjuvant therapy exhibited mutations in genes involved in immune regulation that are also associated with smoking (211). Early-stage LUSC patients also revealed immune marker mutation profiles based on their somatic mutational burdens, which may justify a personalized immunotherapeutic approach to target cancers at early stages of disease progression (247). Immunotherapy had been also employed as an intervention in the adjuvant and neoadjuvant settings, the former of which is based on the premise that immunotherapy will enable host immunity's recognition of the tumor-specific antigens (248–251). In early-stage NSCLC patients, nivolumab administration prior to surgery activated a pathological response in 45% of the tumors studied, which, in turn, increased density of T cell clones in the tumor compartment as well as in the periphery (251). This effect was further intensified by additional blockade of PD-1, whereby T cell activation in the lymph nodes promoted CD8+ T cell infiltration as well as neoantigen-specific T cell clonal expansion in peripheral blood and subsequent infiltration of TILs into tumor beds (251). While the first line treatment for many resectable forms of lung cancer is surgery, advanced-stage tumors hamper surgical intervention. This calls for non-surgical neoadjuvant therapies, including neoadjuvant immunotherapy, which is able to shrink late stage tumors and render them operable, as well as enhance T cell priming in micrometastatic cancer areas, thereby minimizing the chance of recurrence (251). Accordingly, similar studies investigating multiple immune-based neoadjuvant modalities, across larger cohorts and with long-term follow-up, are underway in a number of lung cancers.

### Premalignant Lung Lesions in Animal Models: Field Effect

Because of the difficulty in monitoring lung PMLs early on in human subjects, the majority of our knowledge on the early genomic landscape of lung cancers is derived from experiments in highly pertinent *in vivo* murine models, which are now also being heavily used to understand the potential implication of host immunity in early development of lung cancer. Interrogation of early changes in mice exposed to tobacco-related carcinogen revealed that cytologically-normal airway epithelia harbored prominent and persistent changes signifying a lung “field of injury” (252). The mutagenized airway profiles were pertinent to the step-wise progression into invasive tumors, as well as evolutionarily conserved and evident among cancer-free smokers where the field effect is also prominent (252). Among the significantly modulated gene expression changes was the upregulation of inflammatory markers and the overall activation of aberrant immune signaling pathways, thereby reflecting early immunomodulatory effects in the tobacco carcinogen-exposed airway tissue (252). Using other mouse models with genetically induced *Kras*-mutations, the same variants that are implicated as drivers of human LUAD in smokers, researchers have shown that inhibition of IL-6, whose pleiotropic roles include promoting inflammation as well as growth and differentiation of B and T cells, restricted the development of lung oncogenesis (201, 253). This was mediated by a reduction of M2-type macrophages and an abrogated T-regulatory host cell response, signifying a reprogramming of the TME toward an antitumor microenvironment (201). When treated with mAb targeting IL-6, these mice harbored fewer and smaller lung lesions, less evident lung surface neoplasms, a prominent 46% reduction in the proliferation of tumor cells, and a downregulation of Stat3 activation (201). Further investigation revealed that *Stat3* appears to play diverse roles that are cell-type specific (e.g., epithelial vs. immune cell) as well as gender-specific, whereby female mice with additional deletion of *Stat3* conferred antitumor immunity, compared to a *Stat3*-deletion-mediated pro-tumor immune responses in male littermates (235).

### COPD Markers as Surrogate Tools for Early Management of Lung Cancer

While animal models provide valuable mechanistic insights which can be validated in pertinent cohorts as a means of biological interpretation of response to therapy, there have been other parallels and precursors besides the elusive PMLs, which, if interpreted with caution, can direct the search for immune-based biomarkers for early detection of lung cancer. One prominent example is COPD, a chronic disease of the lung characterized by inflammation of the epithelial airway and alveoli and driven by the exposure to cigarette smoke (201, 240). Several studies have shown a tightly knit association, sometimes causal, between COPD and lung cancer particularly among smokers. First, COPD is evident among 40–70% of lung cancer patients (254). Second, smokers with COPD have an increased risk of lung cancer (3- to 10-fold) compared to smokers without COPD, whereby the inflammation persists and lung function continues to deteriorate as does the increased risk of lung cancer even after smoking cessation (201, 240, 255, 256). Furthermore, and similar to lung cancer patients, COPD displays a “field cancerization” effect, whereby normal tissues adjacent to the tumor harbor genetically and molecularly altered cells (e.g., altered by tobacco carcinogen) that are characteristic of the nearby lung tumor itself in a spatially-distributed manner (257).

Evidence that COPD may promote lung cancer in smokers is further supported by studies delineating the immune contexture in COPD patients. Analysis of bronchoalveolar lavage fluid shows elevated levels of TNF-α, IFN-γ, IL-6, IL-8, neutrophils, macrophages, and CD8+ T cells which is suggestive of a Th1-induced inflammatory milieu (190) (see Table 1). A similar pattern of lymphocyte expansion driven by the increased CD4+ T cell clonality is also evident in lung cancer patients with COPD (258). Further, tumors with COPD manifest with an upregulation of TIM-3 and PD-1 checkpoints on lymphocytes and an increased in PD-1+ CD4+ T cells compared to healthy individuals (258). The upregulation of both checkpoints was also evident in mouse models whereby it was associated with an exhausted immune phenotype (258). Consequently, NSCLC patients with a history of COPD had longer progression free survival in response to anti-PD-1 ICB compared to patients with no COPD (258). The aforementioned studies highlight the importance of considering the expression of COPD biomarkers as a prognostic tool for lung cancer screening, early disease detection, and molecular classification of the disease (259).

### Immunogenomics for a Deeper Understanding of PMLs

Much of our understanding of lung preneoplasia has been accelerated by investigating the genomics of nearby normal-appearing cells, or the “field of cancerization.” Earlier work revealed that visually normal airway cells that have been exposed to smoking and are adjacent to lung tumors, including LUADs, carry alterations that are characteristic of the tumors themselves (257, 260). This airway “field of cancerization” is a premalignant *field* surrounding or adjacent to the lung tumors that is enriched with malignant alterations and, thus, provides biological insights into the development of the tumor (257, 260). The field effect encompasses accumulation of multiple mutational features of lung cancer, thereby signifying a spatiotemporal resolution of lung cancer development from the cancerization field (261). Field-associated gradients have been described at the level of somatic point mutations in cancer driver genes such as *EGFR* and *KRAS*, LOH of 3p and 9p chromosomal regions, epigenetic modulation of *CDKN2A*, copy number alterations, in addition to gene expression profiles across tumor tissue and nearby normal-appearing airway of injury (209). Along the same lines, spatial gradients of allelic imbalance (AI) were also revealed when lung parenchyma, matched NSCLC tissue and normal-appearing airway epithelia were assessed for genome-wide AI (209). A prominent spatial gradient of AI was reported with increasing proximity to tumor sites, across multiple tumor-adjacent and distant airway epithelia from early-stage NSCLC patients (209). Genome-wide AI field profiles and their causative chromosomal alterations were also pertinent in atypical adenomatous hyperplasias (AAHs), the earliest known PMLs of LUADs (205). Common chromosomal aberrations had been previously described as early events in AAHs, such as 17p chromosomal arm loss which arises prior to *TP53* mutation (205) (see Table 1). The latter chromosomal arm loss was previously described in Barret's esophagus as a potential biomarker of pre-neoplastic lesions that would give rise to esophageal adenocarcinoma (205). Furthermore, not only are such differential field cancerization profiles pertinent to early stage lung cancer patients following surgery and thus may be associated with disease relapse (262), but they have been also shown to segregate airways in smokers with lung cancer from airways in smokers without cancer (263, 264). Thus, field effects exemplified here by the AI field, can explain how PMLs arise from the field of cancerization and suggest a key role for the accumulation of particular changes (in this case, chromosomal imbalance-driven genomic instability) and selection early in AAH and NSCLC pathogenesis (205). Whether the field carcinogenesis model affects the immune microenvironment as well-remains an intriguing question worth investigating, with promising avenues for lung cancer immune prevention. Such interrogations are now, more than ever, technically approachable using immunogenomic tools in unique cohorts such as the ones described above.

Technical advances in lung cancer screening as well as its expanded implementation have allowed the detection of what was termed as indeterminate pulmonary nodules (IPNs), as well as brought forth the potential of investigating these resected nodules in novel and unique cohorts. IPNs of the lung were found to be histologically elusive, classifying as AAH, adenoma *in situ* (AIS), minimally invasive adenoma (MIA), or even invasive LUAD (265). Using multi-region exome sequencing, researchers investigated the evolutionary trajectory of those IPNs, revealing evidence for a progressive genomic evolution from AAH to AIS, MIA, and LUADs, as well as a selective evolutionary model characterized by the outgrowth of fit subclones and sweep of unfit subclones, during initiation and early progression of lung preneoplasia (265). Further analysis of neoantigens in this dataset may provide valuable insights into the concurrent evolution of the immunogneomic landscape mirroring the progression of PMLs into LUADs.

Indeed, the advent and expansion of immunogenomics approaches to understanding tumor microenvironment has produced evidence, from several seminal reports, that immunomodulation occurs early on in lung cancer pathogenesis (see Table 1). Deep sequencing investigation of AAHs as well as their corresponding longitudinally sampled LUADs revealed increased activation of the pro-tumor immune pathway (Th2: *CCR2, CTLA4*) as well as suppression of the anti-tumor immune pathway (Th1: *IL12A, GZMB*) during the progression of normal lung to AAHs and further to LUADs (191). This was accompanied by inhibition of *IFNG* and *TGFB1* signaling, reduction in inflammatory responses, and increased expression of *CCL2/CCR2, SPP1*, and *CD27* (191). In another cohort, somatic mutations in AAHs that progressed and persisted in LUADs were not only shown to be highly heterogeneous between PMLs of different patients, but they also produced putative progressive neoantigens that were expressed in the earliest pulmonary PMLs and persisted throughout tumor progression (179). Further, the PML neoantigen load correlated with the extent of CD8+ and CD4+ T cell infiltration and upregulation of PD-L1 (179). These findings suggest an early immune recognition of neoepitopes during lung cancer development, which can be leveraged for the future development of immune-based prevention strategies (179). Further evidence that impairment of adaptive immunity and development of an immunosuppressive environment occurs during early stages of lung cancer pathogenesis, comes from single cell sequencing analyses of paired early stage LUADs and non-involved tissue (266). Interestingly, the succession of genomic and molecular changes occurring as tumors progress from non-invasive PMLs is further paralleled and reflected in the TME, whereby Mascaux et al. demarcated the location and timing of the consecutive and co-evolutionary immunogenomic changes occurring as LUSC PMLs progress into invasive tumors (192). Interestingly, activation of anti-tumor immune pathways was evident and uniquely restricted to the earliest low-grade preinvasive regions, while later stages were characterized by negative regulation of the immune system, antigen processing and the presentation of peptide antigens via an overall upregulatory pattern affecting immunosuppressive genes (192). Along the same lines, the progression of LUSC PMLs, also known as carcinoma *in situ* (CIS), was shown to be predictable using genetic and immune clues. In one report, authors focused on longitudinal CIS biopsies that either progressed to form LUSC or regressed and regained a normal appearance (267). After profiling the genomic, transcriptomic, and epigenomic landscape of those PMLs, the predictive value of specific gene-expression signatures, DNA methylation patterns and copy number alterations was confirmed for specific genes, which could be used to accurately determine the probability of CIS progression (267). Additionally, immune-specific subtypes of bronchial PMLs, LUSC precursors preceding CIS, were shown to correlate with their fate (268). Those that progressed into persistent lesions and henceforth LUSCs were characterized by downregulation of genes involved in interferon signaling and T cell-mediated immunity in comparison to regressive lesions, thus highlighting a dynamic interplay between epithelial and immune pathways early on in premalignant lesions (268). Taken together, these immunogenomics-based findings can provide strong evidence for the interplay between molecular aberrations and immune contexture early on in non-invasive lesions, providing a clinical window of opportunity to derive early detection, prognostic as well as prediction markers for better patient stratification and personalized immune prevention strategies in high risk individuals.

## Conclusion

Empirical evidence supports a pivotal role for the immune microenvironment in modulating tumor biology at early stages in carcinogenesis. A current growing interest is therefore to define whether PMLs are indeed targetable by the various modalities of immunotherapy, an endeavor which has indirectly expanded our knowledge of the immune contexture in lung cancer. Furthermore, while lung PML characterization lends strong evidence to a spatiotemporal evolutionary model characterized by significant and progressive genomic alterations and tumor heterogeneity before the appearance of any overt signs of malignancy, we still do not know whether the surrounding tumor immune microenvironment evolves in a similar and parallel pattern. A deeper scrutiny of PML microenvironment is also now possible when applying the high-resolution and multidimensional genetic, molecular, and cellular immunogenomic techniques probed in late stage cancers, to lung PMLs and their surrounding microenvironment. Not only will the anticipated findings add to the breadth of the currently known map of lung cancer evolution, but it may also challenge us to re-evaluate certain concepts, particularly since immunogenomic signatures of the lung are operative in PMLs and may thus dynamically modulate classically described molecular aberrations (269, 270). Pan-institutional efforts such as pre-cancer atlases are heavily involved in supporting such research directions, by coordinating access to clinical data as well as standardizing research directed toward constructing comprehensive and multidimensional human tumor atlases. Focusing on conditions that are likely to become cancers, such efforts are promising to unveil immune-based biomarkers in lung PMLs (e.g., by characterizing immune infiltrates) and to unravel potential immune interventional strategies at the earliest detectable stages of lung cancer. Resolving transcriptional and immune profiles at the single-cell level, for example of dissociated tumors, is now providing novel insights into intra-tumor heterogeneity, evolution, as well as the pre-existing immunity and its crosstalk with tumor initiation, progression, and response to immunotherapy (266, 271–274). Future directions aimed at understanding the premalignant immune biology of various tumors promise to reveal unprecedented states of tumor plasticity, heterogeneity, and diversity of lymphoid and myeloid cell types in lesions, to harvest potential biomarkers for immune-based treatment of this fatal disease.

## Author Contributions

SS, HZ, AS, and HK conceived the study. SS, HZ, ZR, AS, and HK wrote the manuscript. All authors reviewed the draft manuscript and approved the final manuscript.

### Conflict of Interest

The authors declare that the research was conducted in the absence of any commercial or financial relationships that could be construed as a potential conflict of interest.

## Abbreviations

AIS: Adenoma *in situ*
ACT: Adoptive cell therapy
AI: Allelic imbalance
ADC: Antibody-drug conjugate
APC: Antigen presenting cell
AAH: Atypical adenomatous hyperplasia
BiTEs: Bi-specific T cell-engaging antibodies
CIS: Carcinoma *in situ*
CAR: Chimeric antigen receptor
COPD: Chronic obstructive pulmonary disease
DC: Dendritic cell
FDA: Food and Drug Administration
ICB: Immune checkpoint blockade
IPN: Indeterminate pulmonary nodule
IFN: Interferon
IL: Interleukin
LOH: Loss of heterozygosity
LUAD: Lung adenocarcinoma
LUSC: Lung squamous cell carcinoma
LAK: Lymphokine activated cell
MHC: Major histocompatibility complex
MSI: Microsatellite instability
MIA: Minimally invasive adenoma
mAb: Monoclonal antibody
NSCLC: Non-small cell lung cancer
OV: Oncolytic virus
OPL: Oral premalignant leukoplakia
PML: Premalignant lesion
T reg: Regulatory T cell
SCLC: Small cell lung cancer
TCR: T cell receptor
TLR: Toll-like receptor
TAA: Tumor associated antigens
TIL: Tumor infiltrating lymphocyte
TME: Tumor microenvironment
TMB: Tumor mutational burden
TSA: Tumor specific antigens.
